# Intratissue percutaneous electrolysis and deep dry needling compared to a standard physiotherapy protocol in the treatment of whiplash syndrome: study protocol for a randomized controlled trial

**DOI:** 10.3389/fresc.2025.1670603

**Published:** 2025-11-03

**Authors:** Rocío Fernández-Navarro, María Benito-de-Pedro, Francisco-Manuel Navarro Reyes, Jorge Moreno-López, María-José Estebanez-Pérez, José-Manuel Pastora-Bernal

**Affiliations:** ^1^ Grupo de Investigación en Fisioterapia y Salud (FYSA), Departamento de Fisioterapia, Facultad de Ciencias de la Salud-HM Hospitales, Universidad Camilo José Cela, Villanueva de la Cañada, Madrid, Spain; ^2^ Instituto de Investigación Sanitaria Hospitales HM, Madrid, Spain; ^3^ Department of Physiotherapy, University Hospital of Melilla, Ingesa Melilla, Melilla, Spain; ^4^ Department of Physiotherapy, Faculty of Health Sciences of Melilla, University of Granada, Melilla, Spain; ^5^ Department of Physiotherapy, Faculty of Health Sciences, University of Málaga, Málaga, Spain

**Keywords:** percutaneous electrolysis, dry needling, whiplash, myofascial pain syndrome, myofascial trigger point, intrafibrillar blood flow and elasticity of muscle fibers

## Abstract

**Background:**

Whiplash syndrome is one of the most frequent consequences of traumatic pathology caused traffic accidents. The acceleration-deceleration mechanism of energy transmitted to the neck causes abnormal maneuvers in the area and muscle pain. The most common form of muscle pain in the neck and head is myofascial pain syndrome, caused by myofascial trigger points, these being clinically defined as painful, sensitive and hyperirritable nodules that are located on tense muscle bands. These painful nodules are treated with different physiotherapy techniques, there being no international consensus regarding their diagnosis and recommended interventions.

**Objective:**

The primary objective of this study is to evaluate the effectiveness of percutaneous intratissue electrolysis (EPI) on intrafibrillar blood flow and muscle fiber elasticity compared to deep dry needling (DN) and standard physiotherapy. Secondary objectives include the assessment of perceived pain, disability, and clinical outcomes at follow-up.

**Methods:**

This single-blind randomized clinical trial will be conducted in patients residing in Melilla with whiplash syndrome due to traffic accidents. Participants will receive interventions targeting the sternocleidomastoid and/or levator scapulae muscles. Assessments will be conducted at baseline, after four weeks of intervention, and at three months post-intervention to evaluate medium-term effects. Our hypothesis is that EPI will produce greater improvements in the study variables compared to DN and standard physiotherapy.

**Discussion:**

Percutaneous electrolysis has shown positive clinical effects in various musculoskeletal pathologies; however, its impact on intrafibrillar blood flow and muscle fiber elasticity remains unexplored. This study aims to provide reference clinical data on these physiological outcomes and compare the effects of invasive vs. standard physiotherapy interventions, supporting the development of evidence-based protocols for whiplash-associated disorders.

**Clinical Trial Registration:**

NCT06938425.

## 1 Introduction

Whiplash syndrome (WAD) is a common soft tissue injury of the neck caused by forced flexion-extension movements, usually after traffic accidents (1–3).

WAD caused mainly by vehicle collisions, has an annual incidence in Western countries of between 200 and 300 cases per 100,000 inhabitants (4). It is the most frequent traumatic pathology in the forensic field, with a high incidence in Spain, with the Autonomous City of Melilla standing out (5–10). Clinical manifestations may not be immediate, and between 10%–42% of cases evolve into chronicity (2, 11). Around 16% are left with severe and persistent disability (12). Diagnosis is primarily clinical, as imaging tests are often inconclusive (13, 14). Likewise, the literature indicates that psychological, social, and contextual factors influence the transition to chronicity, underscoring the need for an early and interdisciplinary approach (15).

Myofascial pain is a frequent complication of WAD, associated with the presence of myofascial trigger points (MTrPs), responsible for cervical and cranial pain, with a prevalence that can reach 93% in some populations (16–21). Myofascial pain syndrome (MPS) is characterized by the presence of hyperirritable nodules in tight muscle bands that generate local or referred pain and muscle dysfunction (21, 22). Its pathophysiology involves abnormal stimulation of sensory nerves and excessive release of acetylcholine, resulting in persistent contractions and pain (23, 24). PGMs commonly appear in muscles such as the upper trapezius, sternocleidomastoid and levator scapulae, being frequent in SLC (25–34).

Diagnosis is based on clinical criteria such as spontaneous pain, presence of tight band, tender point and referred pain, and can be complemented with electromyography, ultrasonography, elastography and other techniques (35–45). In addition, factors such as hormonal, nutritional and postural deficiencies may perpetuate the dysfunction (46).

The treatment of WAD has evolved away from the use of drugs such as analgesics, anti-inflammatory drugs and muscle relaxants due to their adverse effects and limited efficacy (47, 48). Rest and the use of soft collars are discouraged because they delay recovery and promote complications such as muscle atrophy (47, 49, 50). Currently, early mobilization and supervised exercise are prioritized, although the efficacy of spinal manipulations is still controversial (49).

In the field of physical therapy, there is no single consensus protocol for treating MPS in the context of WAD, although the common goal is to restore muscle function and deactivate MTrPs (51–53). Individualized programs integrating manual therapy, myofascial release, dry needling (DN), therapeutic exercises, and electrotherapy have been shown to be effective (14, 54–56). International clinical guidelines recommend a multimodal approach based on a combination of active and passive techniques, along with self-care (57). A recent meta-analysis confirmed that combined techniques achieve better results in terms of pain reduction and sensitivity improvement (58). Still, more methodological quality is needed to establish a standardized protocol (47, 50, 59–61).

Dry needling (DN) is a technique increasingly used to treat musculoskeletal pain, especially in MTrPs, by inserting a needle without substance, provoking a mechanical stimulus to deactivate it (62–66). It is classified into superficial dry needling (SDN) and deep dry needling (DDN), the latter being the one that goes through the MTrPs and can induce a local spasm response (63, 65, 66). The most recognized techniques are those developed by Hong and Gunn, applied in pathologies of the neck, shoulder and back (67–73). Its indications are focused on the treatment of musculoskeletal pain, with absolute and relative contraindications such as belonephobia, coagulation disorders, pregnancy or immune diseases (74–91). If performed under conditions of asepsis, informed consent and ultrasound guidance, it is a safe technique, although adverse effects such as hematoma, infection or pneumothorax may occur (83, 92–110).

DN acts through the release of endogenous opioids, washout of inflammatory mediators, mechanical destruction of altered muscle fibers, and normalization of sarcomeric length (35, 111–118). The use of 0.30–0.32 mm stainless steel needles adapted to the treated region is recommended (98, 99). Studies suggest that DN can reduce pain and increase pressure threshold in the short term in cases of mechanical neck pain, although its long-term benefits compared with passive stretching are limited (89, 119–122). Some meta-analyses suggest that infiltrations may be more effective than DN in terms of pain reduction, with no significant difference in disability or mobility (123, 124). Currently, the evidence is insufficient to confirm their superiority over placebo (62, 93, 125–127).

Intratissue percutaneous electrolysis (EPI) is an invasive minimally physiotherapy technique that applies galvanic current through a needle to induce a controlled inflammatory response and promote tissue repair. By combining mechanical and electrical stimuli, EPI has demonstrated effectiveness in reducing pain and disability in tendinopathies and other musculoskeletal pain conditions, with favorable outcomes reported in the short, medium, and long term. Moreover, when adverse events are reported, they are generally infrequent and mild, although safety reporting remains limited and should be further standardized in future clinical trials (128–130). Designed by Sánchez Ibáñez in 2003, its application requires rigorous asepsis and ultrasound guidance to ensure safety and accuracy (98, 99, 101, 131).

Galvanic current is administered by means of an active cathode (needle) and an anode, with doses determined by Faraday's Law: high intensities and short times for regeneration; low intensities and long times for analgesia (99). It is contraindicated in cases such as unstable epilepsy, open wounds or metallic implants (83). EPI has shown pain reduction and functional improvement with moderate evidence, especially in chronic tendinopathies combined with eccentric exercise (99, 132, 133). However, some studies have found no relevant differences with respect to placebo or DN (134–137). It has also been evaluated in conditions such as lateral epicondylalgia, temporomandibular dysfunctions and rotator cuff pain, where it has outperformed DN in some cases (130, 133, 138–140), although more research is needed to validate these results due to methodological variability (85, 130, 133, 141–147).

The primary objective of this research is to evaluate the efficacy of two physiotherapeutic interventions - percutaneous intratissue electrolysis (PIE) and deep dry needling (DDN) - in adults with WAD after traffic accidents and to see if there is an improvement in intrafibrillar blood flow and muscle fiber elasticity compared to a standard physiotherapy protocol. Secondary objectives include the evaluation and analysis of perceived pain, disability, and long-term variable clinical outcomes.

## 2 Methods

### 2.1 Study design

**Figure 1:**
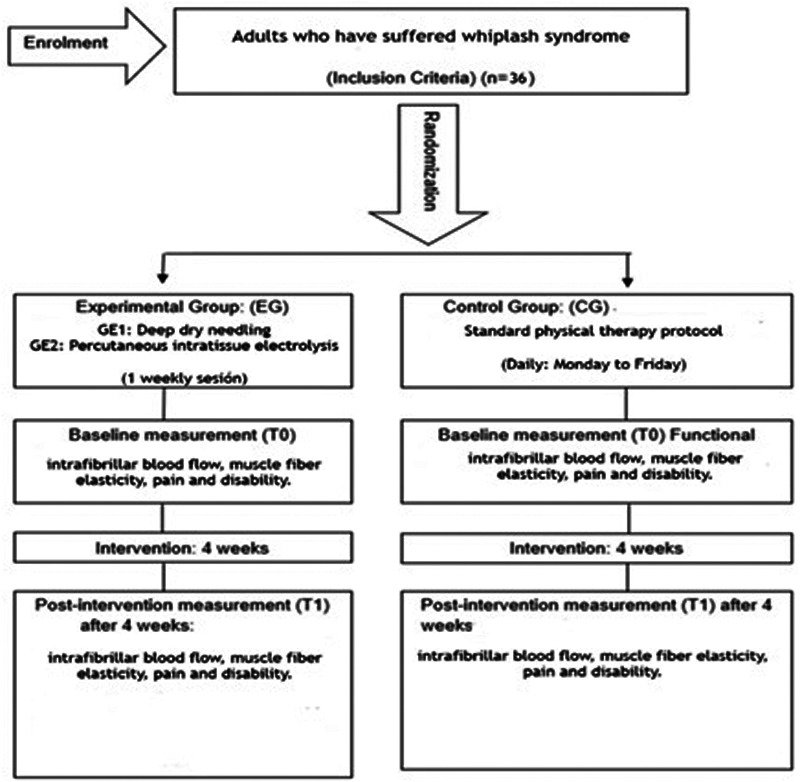
Flow chart of the study design.

This research will be conducted as a single-blind randomized clinical trial in patients residing in the Autonomous City of Melilla with whiplash syndrome following a traffic accident. This research uses the Standards for Quality Improvement and Reporting Excellence (SQUIRE) guidelines (148) and will be conducted according to the Consolidated Standards for Reporting of Clinical Trials (CONSORT) criteria (149). The Standard Protocol for Interventional Trials (SPIRIT) Item Recommendations checklist has been added as Supplementary Material (150). A flow chart of the study design is shown in Figure 1.

### 2.2 Study sample

The subjects will be recruited from a health center adhered to the agreement on health care derived from traffic accidents [Unión Española de Entidades Aseguradoras y Reaseguradoras (UNESPA) agreement] belonging to the Autonomous City of Melilla. The following inclusion and exclusion criteria will be used to select the sample.

The inclusion criteria for this study will be as follows:

- The study includes adults with whiplash syndrome (CIE-10- code S13.4) with pain in the cervical area after a traffic accident. Subjects will have to be treated in hospitals or private centers and referred to rehabilitation.
- Patients must reside or stay in the Autonomous Community of Melilla during the intervention phase.
- Subjects will have the presence of neck pain after suffering a traffic accident and the presence of at least one active trigger point in the sternocleidomastoid and/or levator scapulae muscles.
- All subjects suffering a traffic accident and having a score of 5 or higher on the numerical scale (EN) of pain assessment.

It will be a reason for exclusion:

- Patients doing anticoagulant treatment.
- Patients who have suffered previous cervical trauma or who have undergone surgery in the last year.
- Subjects who present any type of alteration (skin or infection, sensitivity or pain perception).
- Subjects with central or peripheral nervous system affectation.
- Patients who have problems with the material: allergy to metal, belonephobia (fear of needles).
- Pregnant women.

### 2.3 Sample size

To date, there are no studies reporting on the use of deep dry needling and/or percutaneous intratissue electrolysis in patients who have suffered from whiplash syndrome, so that this randomized, blinded clinical trial will provide evidence for the effect size.

To calculate the population size, we used data published by the Directorate General of Traffic (DGT), updated in 2020, which identified 242 patients who were victims of traffic accidents in the city of Melilla. The Al-Therapy Statistics tool will be used for the sample calculation (https://www.ai-therapy.com/psychology-statistics/sample-size-calculator). Therefore, we will use a sample size of (*n* = 36) with a proportional distribution for each study group (GE1 group = 12 + GE2 group = 12 + Control group = 12) with an expected loss ratio (R = 15%) and a confidence interval (CI = 95%), this information is expanded in the Supplementary Material.

However, this calculation does not provide sufficient statistical power. A more robust estimation can be derived from validated clinical parameters reported in the literature: a minimum clinically important difference (MCID) of 2 points on the Numeric Rating Scale (NRS) for pain (151–153), a standard deviation of approximately 2.5 (154), and a pre–post correlation ranging between 0.5 and 0.7 (155, 156). Based on these assumptions, the required sample size would range from 20 to 31 participants per group (60–93 in total) to ensure adequate power.

Since the present study will include only 36 participants, it should be explicitly characterized as a pilot randomized controlled trial, designed to assess feasibility, refine procedures, and provide preliminary effect size estimates to guide the design of a fully powered clinical trial.

For the development of this research, a non-probabilistic intentional sampling will be used for the convenience of the study, due to the characteristics of the subjects to be studied. Strategies to ensure adequate inclusion of participants who meet the target sample size include collaboration with a health center affiliated with the UNESPA agreement on healthcare resulting from traffic accidents, rehabilitation physicians, and physiotherapy teams.

Collaborators will be informed about the study's characteristics during personal interviews and a project presentation. We strive to ensure that patient recruitment reflects sociodemographic diversity in terms of social origin, gender, ethnicity, and education, tailored to the specific characteristics of the reference population in Melilla and with prior information regarding compliance with data protection laws.

### 2.4 Randomization

Before patient inclusion, the research team will generate the allocation sequence and randomly assign patients consecutively to GE1, GE2, and the CG. A computerized random number generator will be used. Each participant will be treated separately to prevent any exchange of study information. The nature of the intervention in both groups does not allow blinding of patients and physiotherapists. It is therefore a single-blind study, where the evaluator does not know the nature of the intervention. The evaluator was unaware of the study objectives and the randomized distribution of patients to study groups, and he did not have access to the randomization sequence.

Before patient inclusion, the research team will generate the allocation sequence and randomly assign patients consecutively to GE1, GE2, and the CG. A computerized random number generator will be used. Each participant will be treated separately to prevent any exchange of study information. The nature of the intervention in both groups does not allow blinding of patients and physiotherapists. It is therefore a single-blind study, where the evaluator does not know the nature of the intervention. The evaluator was unaware of the study objectives and the randomized distribution of patients to study groups, and did not have access to the randomization sequence. The evaluator was blinded to treatment allocation throughout the study. Participants were explicitly instructed not to disclose their assigned intervention to the evaluator; treatments and assessments were performed by different staff in separate locations; and any potential unblinding events were systematically logged. At the end of follow-up, the evaluator recorded, for each participant, their guess of allocation (active/sham/don't know), allowing for a quantitative evaluation of blinding success.

### 2.5 Intervention

Study subjects are randomly assigned to one of the following three groups: Experimental Group 1 (EG1), Experimental Group 2 (EG2) or the Control Group (CG) through a computer randomization system using the statistical program Epidat V4.2.

Once the subjects have been informed of the entire study procedure and have signed the informed consent form (Annexes I, II) this information is expanded in the Supplementary Material, they will be evaluated before starting treatment. Subjects will receive an initial evaluation based on clinical parameters and follow-up medical records. The data will be collected by an evaluator and integrated into our research databases using an Excel spreadsheet.

Both experimental groups will receive an invasive physiotherapy protocol (one weekly session) applied to the sternocleidomastoid and/or levator scapulae muscles, in addition to the standard physiotherapy program. The intervention will be conducted over four weeks, with a total of 20 sessions (five per week), following rehabilitation medicine guidelines for this condition. This standardization ensures consistency in treatment exposure and enhances reproducibility of the protocol.

The therapeutic exercise program will be delivered under the supervision of trained physiotherapists. Exercises will be performed in progressive phases, with adjustments in intensity, resistance, and volume according to predefined progression criteria. Details of the exercise program are summarized in Table 1.

**Table 1:** Relationship between standard physiotherapy procedure and intervention.

Intervention procedure	Physiotherapy intervention	Description	Duration/Repetitions	Frequency	Subjects
Experimental group	EPI	Hong technique on localized MTrPs (ECOM and/or Levator scapulae)	1× week	1 day for 4 weeks	GE_1_
	Dry needling	Hong technique on localized MTrPs (ECOM and/or Levator scapulae)	1× week	1 day for 4 weeks	GE_2_
Standard physiotherapy procedure (GE + GC)	Thermotherapy + analgesic electrotherapy	Microwave or thermotherapy techniques combined with TENS.	15 min	5 days for 4 weeks	GE + GC
	Massage therapy	Friction maneuvers, kneading, and manual stretching of the muscle and affected area.	20 min	5 days for 4 weeks	GE + GC
	Manual therapy	- Deep friction massage - Contraction- relaxation technique passive - Stretching and self-stretching	10 min	5 days for 4 weeks	GE + GC
			2–3 repetitions		
	Active mobility joint range	- Free active mobility exercises in all planes of motion of the affected joints	2–3 repetitions per movement	5 days for 4 weeks	GE + GC

**Figure 2:**
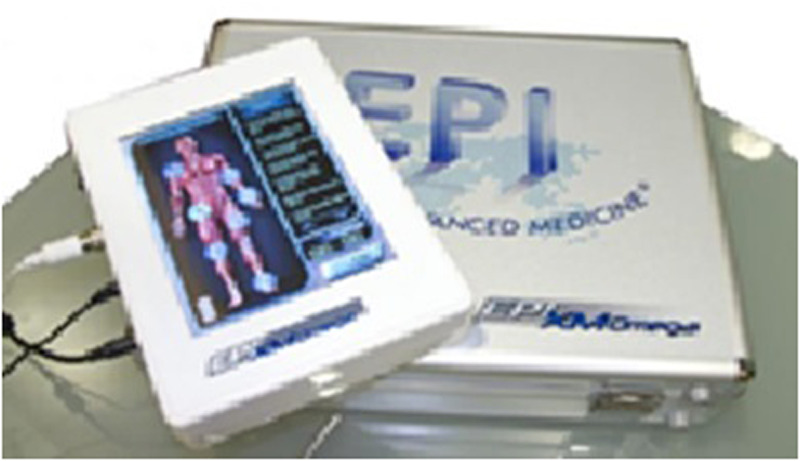
EPI Model: XM Omega.

Currently there are several devices to perform the EPI technique, we will use the EPI XM Omega model for our study (Figure 2). Before performing the technique, the area to be treated will be disinfected and cleaned with 2% chlorhexidine, and gloves will be put on. Next, the areas of hyperalgesia will be located by palpation, and then, using ultrasound, different areas of degeneration will be searched for in that localized point. To apply the current, an intensity of 3 milliamps (mA) will be applied for 5 s according to the established protocol for the technique (130, 132, 133). This method will be performed three times on each target point found. Performing the technique creates an inflammatory response in the tissue, which can last from 5 to 7 days to a maximum of 15 days. After two weeks, it can be confirmed that there is no inflammatory infiltrate at the site of application (130, 144, 147, 157).

In the case of dry needling treatment on a myofascial trigger point located in the sternocleidomastoid and/or levator scapulae muscles of the affected area, the aforementioned asepsis and hygiene measures must also be followed beforehand.

The modified Hong technique (quick entry and exit) is performed, seeking a local spasm response—at least one response, indicating that the treatment is more effective than without it (62). Once the needle is withdrawn, pressure should be applied to the puncture site where the technique was performed for 1–2 min to prevent bruising. This is done as post-needle care to prevent post-needle pain and promote hemostasis.

The procedure at the control group will include standard physical therapy following the progression criteria based on clinical guidelines for patients with whiplash syndrome (14), treating the sternocleidomastoid and/or levator scapulae muscles with a frequency of 5 weekly sessions for 4 weeks. The two professionals performing the procedure are professionals with more than ten years of experience in invasive techniques. Before the start of the trial, the therapists participated in a training and protocol standardization session to ensure consistency in the application of the procedures. Treatment fidelity measures were implemented throughout the study, and any protocol deviations were systematically recorded. The professionals and evaluators are different.

### 2.6 Outcomes measures and tools

The initial assessment includes a clinical history-taking interview. We will strive to ensure the sample data are homogeneous at the start of the study, ensuring there are no significant differences in demographic, medical, and other variables at the outset.

#### 2.6.1 Primary outcome measures

##### 2.6.1.1 Intrafibrillar blood flow

After suffering a traffic accident and consequently suffering from CLS, the cervical vertebrae may become blocked. This leads to a slight lack of blood supply to the brain, compromising its vascularization due to the compression of the vertebral artery, producing a feeling of instability (158). The arteries considered most important in the neck, according to the study by Martinez Medina (159), since blood flow to the brain occurs primarily through two arteries: the vertebral artery, located in the posterior region, and the carotid artery, located in the middle and anterior part of the neck. Compression of these main arteries can cause cervical dizziness. These are associated with various symptoms such as dizziness, tension in the neck, nausea, vertigo, and photophobia or sensitivity to light (158).

The vertebral artery may be compressed in some areas along its path to the skull or at the entrance to it, where it must bend to avoid the occipital condyle. If the position of this condyle varies due to the tension of the cervical muscles, it is obvious that the artery is slightly compressed. As for the carotid artery, it may be compressed in its fascial sheath common to that of the anterior cervical muscles or at the entrance to the skull (158).

Ultrasonography, also known as musculoskeletal ultrasound, is an important diagnostic tool that has evolved both technologically and in terms of understanding pathologies affecting soft tissues such as the skin, muscles, bursae, tendons, and ligaments, among others (160).

In this study, the term intrafibrillar blood flow is used in an operational sense to describe local muscle microcirculation assessed indirectly through Doppler ultrasound. Although this imaging technique does not measure blood flow within muscle fibers *per se*, it provides information on the perfusion of small-caliber vessels adjacent to the fibers. These measures are interpreted as indirect indicators of the metabolic state of the tissue and its capacity for recovery. By adopting this approach, we aim to complement traditional patient-reported outcomes with objective physiological parameters that may reflect tissue-level healing processes.

This type of examination is performed on a digital device with a high-frequency linear array transducer (47). Furthermore, color Doppler can be used to assess small vessel hypervascularity (slow flow) in the inflamed synovial tissue of the studied structures (160).

This instrument is used to measure intrafibrillar blood flow, which is the volume of blood pumped in a unit of time. Measurements are expressed in milliliters per second (ml/s) or liters per minute (l/min). In turn, the speed at which the flow moves, that is, the distance traveled in the unit of time, is measured in centimeters per second (cm/s) or meters per second (m/s) (161).

##### 2.6.1.2 Muscle fiber elasticity

There are three types of muscle: skeletal muscle, cardiac muscle, and smooth muscle, focusing on skeletal muscle.

Its unique physiology determines a series of characteristics unique to this tissue. Its most interesting property is the ability to contract under electrical stimulation derived from voluntary nerve control, which leads to changes in its structure and mechanical properties (162). Over the last century, numerous investigations have been conducted on changes in the mechanical properties of muscle tissue using elastography, specifically quantitative elastography (Shear-Wave: SWE). Recent literature has shown promising results in the evaluation of various traumatic and pathological conditions in various musculoskeletal tissues, including muscles, ligaments, and nerves (163).

Elastography or sonoelastography (SE) uses an electronic device to generate an acoustic pulse that produces tissue deformation through a transverse shear wave (163). This device is an imaging technique based on the differentiation of the elasticity of healthy tissue compared to pathological tissue, which can be measured based on the tension applied to the tissue. The elastic properties of soft tissue depend on their molecular conformation and structural organization, both microscopically and macroscopically (163). It also allows the characterization of the mechanical and intrinsic properties of muscle tissue: edema, atrophy, and fatty infiltration (163). In this way, it measures the velocity (m/s) of the waves generated by the acoustic pulse at all points in the tissue studied, which allows the stiffness and elasticity of the tissue to be expressed in units of pressure (kilopascals) (163).

Elastography is obtained when a color parametric image expressing the velocity of tissue deformity is superimposed on the grayscale ultrasound anatomical image. These tissues tend to develop greater deformity, while those with greater stiffness show less deformity (163). More technically, elastography is an imaging technique that distinguishes the stiffness of affected tissue, always comparing it with normal tissue, and codes it in color.

The technique displays two distinct colors: red and blue. Red indicates a stiffer tissue, while blue indicates a softer tissue. Yellow or green indicates a tissue with intermediate characteristics (163, 164).

#### 2.6.2 Secondary outcomes measures

##### 2.6.2.1 Pain

Pain is a quantitative variable characterized by being a subjective phenomenon, and there are many ways to classify it (165). We will use the numerical rating scale (NRS) (166), which consists of a numbered scale from 1 to 10, where 0 is the absence and 10 is the greatest perceived intensity. The patient will be asked to mark the number that determines the intensity of the symptom.

Another quantitative variable of pain is the pressure pain threshold (PPT) (167). A pressure algometer will be used to measure this variable. This instrument measures “the minimum amount of pressure necessary to cause discomfort at a given point” (168). Measurements are expressed in kg/cm^2^, with the maximum being 10 kg/cm^2^. This instrument has been shown to be valid, reliable, and reproducible for quantifying pain sensitivity in patients with myofascial pain syndrome (169).

We will use these two instruments, since the NRS presents subjective and emotional factors of the patient while the algometer is more quantitative and is associated with nociceptive weakness based on a noxious stimulus (170).

##### 2.6.2.2 Disability

According to the World Health Organization (WHO), the generic term “disability” covers all impairments, limitations in performing activities and participation restrictions, and refers to the negative aspects of the interaction between a person with a health condition and contextual factors of that person such as environmental and personal factors (171). To assess disability, we will use the scale called Whiplash Disability Questionnaire (WDQ) (Annex III); this information is expanded in the Supplementary Material. It is the first instrument designed by Hoving et al. (172) for patients with whiplash syndrome, based on the neck disability index (NDI), which includes items related to areas of life, personal care and work, as well as those related to pain perception. To measure the degree of disability in whiplash syndrome of the participants, we will use this questionnaire in its Spanish version (173). This questionnaire consists of 13 items, scored from 0 to 10 points, where 0 corresponds to no pain/not at all and 10 corresponds to I can't/Always.

A summary of the research outcomes and measurement instruments is shown in Table 2.

**Table 2:** Relationship between variables and measuring instruments.

Variables	Instrument/Tool	Type
Intrafibrillar blood Flow	Doppler ultrasound examination	ml/sg or L/min
Muscle fiber elasticity	Elastography	Pressure unit (kilopascals)
Pain	Numeric scale (NS)	Numeric
	Pressure algometer	Kg/s
Disability	Whiplash Disability Questionnaire (WDQ)	Numeric

### 2.7 Data collection procedure, monitoring, and management

During data collection, the information will be anonymized, and all data from the clinical history, anamnesis, and the various study variables will be compiled. Once all the information has been gathered, an individual palpatory assessment of the sternocleidomastoid and/or levator scapulae muscles will be performed, along with a set of tools and instruments for measuring variables.

The procedure for taking variable measurements and evaluations will be carried out before the start of the program (Pre-Test), four weeks after the completion of the intervention (Post-Test).

Both the initial assessment (Pre-Test) and the four-week measurements (Post-Test) will be performed by an evaluator unaware of the nature of the intervention, at a designated location, with easy access for the patient and prior appointment confirmation.

The data will be added to the database created for this purpose and managed by the principal investigator. A data collection notebook will be used as supporting material and will be used for statistical analysis.

The results of the research will be presented as a summary of the outcome measures, along with the estimated effect size and its precision. Statistical analysis will be performed according to the intention-to-treat principle using SPSS software, with statistical significance set at *p* < 0.05. Descriptive statistical analysis of the different variables will be performed. Results will be evaluated by comparing differences between groups using linear mixed model statistics and the *t*-test to test the hypothesis that group means are or are not significantly different from each other.

## 3 Discussion

Musculoskeletal pain is the clinical symptom with the highest demand for medical attention and the leading cause of disability worldwide (174). The anatomical area with the greatest localized pain and most affected is the cervical region (175), frequently accompanied by the head region (176, 177). One of the most common causes of neck pain related to the development of MTrPs within the musculoskeletal system, which are known as small foci of hyperalgesia characterized by producing localized pain (176).

In this study, we will compare different treatment approaches such as electrolysis, dry needling, and standard treatment in a population that, in recent years, has shown a high prevalence of neck pain, some of which has led to sick leave, related to traffic accidents. This population, previously diagnosed with myofascial pain syndrome associated with their neck pain, received treatment at the MTrP of the sternocleidomastoid and/or levator scapulae muscles, a structure associated with this syndrome and pain in the craniocervical region (50, 178, 179). Therefore, whiplash syndrome is considered a key clinical sign to address (5, 6, 57).

Within the perspective of the evolution of WAD recovery, studies have been conducted on the neck muscles. These articles show a tendency toward muscle degeneration that occurs shortly after the injury. This muscle degeneration has risk predictors that include advanced age, pain-related disability, and post-traumatic stress disorder. These changes may appear a few weeks after the accident, but only in patients who show poor recovery (180–182).

A law has even been passed in Spain, Law 35/2015, which is quite restrictive in its consideration of whiplash syndrome, and at the same time is specific in requiring a conclusive medical report, so that the consequences of a WAD are assessed and compensation is not simply awarded as temporary injuries (183).

There are studies on the most important prognostic factors in these patients. Other variables have been linked to an unfavorable outcome of WAD, such as ongoing legal claims, pending compensation, sick leave, a history of psychiatric illness, low educational level, higher levels of somatization and/or sleep difficulties, and pain intensity (5). According to a systematic review by Serrano D et al. In this article, they conclude that future research is urgently needed to delve deeper into the pathophysiology of WAD, as it affects a large portion of the population and is a frequent cause of lawsuits, compensation, healthcare costs, and prolonged pain and disability for some patients, who are sometimes unfairly classified as malingerers (184).

We would like to emphasize that the costs of whiplash, considering those cases in which symptoms become chronic, would be incalculable in the long term.

The results obtained from various studies suggest that a combination of interventions with invasive techniques and standard physical therapy can be effective in improving intrafibrillar blood flow, muscle fiber elasticity, pain, and disability related to whiplash syndrome.

This study protocol represents the first objective to investigate the effectiveness of electrolysis or deep dry needling combined with standard physical therapy treatment for whiplash syndrome. Therefore, our objective of this research is to gather information and knowledge on the possibility of incorporating invasive techniques such as EPI or DN into a standard physical therapy program, in order to identify new intervention opportunities that could benefit patients. The effectiveness of this intervention and patient satisfaction with recovery will serve as a primary indicator of whether the study provides additional evidence supporting the use of invasive techniques as an effective tool for patients who have suffered whiplash.

Future lines of research may involve the development of clinical trials with larger sample sizes. The feasibility of this pilot study will serve as a basis for future research, in which we will support the basic design of this study, expand the sample size, and attempt to standardize intervention protocols across different healthcare centers.
