# Supplementary material for: Intratissue percutaneous electrolysis and deep dry needling compared to a standard physiotherapy protocol in the treatment of whiplash syndrome: study protocol for a randomized controlled trial
Source: Front Rehabil Sci. 2025 Nov 3;6:1670603. doi: 10.3389/fresc.2025.1670603 (PMC12620460; doi:10.3389/fresc.2025.1670603)
Supplement: Supplementary file 1 [file Datasheet1.pdf]

Supplementary Material

Supplementary Figure 1. MINIMAL SAMPLE SIZE

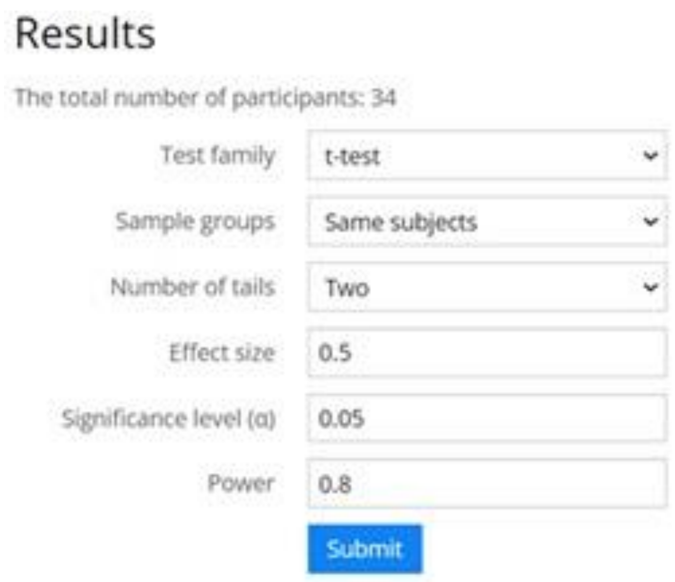

Annex I. **Information Prior to Informed Consent**

<u>Patient Background Information</u>

WHY ME? Because you are over 18 years old and have recently been selected by _ _ _ _ _ _. From what the Medical team has seen that it can benefit from the resources offered by this research project.

WHAT DOES THE PROJECT CONSIST OF? An accepted intervention is applied and validated by the scientific community. Similar studies have shown that it is not lower than other interventions and in similar pathologies. We want to check its effectiveness in certain patients.

WHAT HAPPENS IF I DECIDE TO ALSO APPLY OTHER TYPES OF MEASURES?

Your advisor during the research period will inform you what type of medical care you will receive and how you should report any other type of intervention that may be performed. influence the results.

AND AFTER THE 4 WEEKS OF INTERVENTION, WHAT HAPPENS?

Your advisor during the investigation period will inform you of the recommendations to continue once the intervention is completed.

IF I DON'T FEEL LIKE IT, I CAN'T, I DON'T WANT TO CONTINUE STUDYING, WHAT

HAPPENS?

You can decide at any time whether to include it, as well as your exclusion from the project without further communication and without giving explanations if you so wish.

WHAT GUARANTEES DO I HAVE IF I DECIDE TO PARTICIPATE?

Firstly, the Project has been approved by the Research Ethics Committee of ……………., so the interventions to be carried out have the maximum guarantees required by good professional practice and safety. In addition, the monitoring will be exhaustive by part of the researchers with more than 10 years of professional experience.

Annex II. Informed Consent

Informed Consent – Written Consent of the Patient

I (Name and surname):

....………………………..

1. I declare that I have read the Patient Information Sheet that accompanies this consent.
2. I was able to ask questions about the study. All questions were answered to my complete satisfaction.
3. I have spoken with the health professional who provided the information: ……………………….
4. I understand that my participation is voluntary and I am free to participate or not in the study.
5. I have been informed that all data obtained in this study will be confidential and will be treated as established by the Organic Law on Protection of Personal Data 15/99.
6. I understand that I may withdraw from the study:
   - Whenever you want
   - Without having to give explanations
   - Without this affecting my medical care

I freely give my consent to participate in the project entitled

GIVE ☐

I DON'T GIVE ☐

Patient's signature

Signature of the reporting healthcare professional

Name and surname: ……………

Date: ………………………

Name and surname: ………..

Date: ………………………
